# Lemon Dietary Fibre-Based Powder as a Promising Ingredient for the Food Industry: Enhancing *Mortadella* Nutritional Quality

**DOI:** 10.3390/foods14101693

**Published:** 2025-05-10

**Authors:** Daniela Magalhães, Cristina V. Rodrigues, Carmen Botella-Martinez, Nuria Muñoz-Tebar, José Angel Pérez-Álvarez, Manuel Viuda-Martos, Paula Teixeira, Manuela Pintado

**Affiliations:** 1 CBQF—Centro de Biotecnologia e Química Fina—Laboratório Associado, Escola Superior de Biotecnologia, Universidade Católica Portuguesa, Rua Diogo Botelho 1327, 4169-005 Porto, Portugal; dmagalhaes@ucp.pt (D.M.); civrodrigues@ucp.pt (C.V.R.); pcteixeira@ucp.pt (P.T.); 2 IPOA Research Group, Institute on Agri-Food and Agri-Environmental Research and Innovation (CIAGRO-UMH), Miguel Hernandez University of Elche, Carretera de Beniel, km 3.2, 03312 Orihuela, Alicante, Spain; c.botella@umh.es (C.B.-M.); nmunoz@umh.es (N.M.-T.); ja.perez@umh.es (J.A.P.-Á.); mviuda@umh.es (M.V.-M.)

**Keywords:** dietary fibre, lemon co-products, bioactive properties, sensorial analysis, functional meat foods, nutritional value

## Abstract

Lemon co-products are valuable due to their high dietary fibre, making them significant for valorisation. This research aimed to characterise an innovative lemon dietary fibre (LDF) obtained through integrated extraction (of essential oil, phenolic compounds (PCs), and pectin) by evaluating its chemical, physicochemical, structural, techno-functional, total phenolic content, and antioxidant and antibacterial properties. The effects of incorporating LDF (3% and 6%) into *mortadella*, a bologna-type sausage, on chemical, physicochemical, technological, and sensory properties were analysed. LDF exhibited a total dietary fibre content of 85.79%, mainly insoluble (52.55%). Hesperidin (89.97–894.44 mg/100 g DW) and eriocitrin (68.75–146.35 mg/100 g DW) were the major free PCs. The major bound PCs were vanillin (5.90–9.16 mg/100 g DW) and apigenin-7-O-glucoside (8.82 mg/100 g DW). This functional ingredient demonstrated antioxidant and antibacterial activity. LDF significantly influenced *mortadella*’s colour, texture, and mineral composition. Higher levels of LDF result in a paler colour and increased hardness and contribute to reducing sodium levels of the final product. It also decreased residual nitrite levels, although this reduction was followed by a slight increase in lipid oxidation, which remained below the rancidity threshold (≥1.0), ensuring acceptable product quality. Sensory evaluation revealed positive feedback, favouring the 3% LDF formulation.

## 1. Introduction

The search for innovative and sustainable ingredients remains a key focus in the constantly evolving food industry. Since consumer preferences have been shifting towards healthier and environmentally conscious choices, the valorisation of lemon co-products addresses waste management concerns and offers a valuable source of functional ingredients [1]. Citrus juice industries generate a significant amount of co-products, which are mainly used for animal feed. These wastes account for 50% of the original whole fruit and mainly comprise peels (albedo and flavedo), almost one-fourth of the whole fruit mass, seeds, and fruit pulp after juice and essential oil extraction. However, because of their high fibre content, they can be used as a good source of dietary fibre (DF) [2,3]. DF is a complex mixture of polysaccharides, which can be classified into two categories based on water solubility: soluble (SDF) and insoluble dietary fibre (IDF). Soluble dietary fibre includes gums, pectin, glucans, and some biological and synthetic polysaccharides, while IDFs include cellulose, hemicellulose, lignin, and non-cellulosic polysaccharides [4,5].

Lemon fibres are known for their exceptional quality, attributed to their low caloric content and abundant fibre content, leading to favourable functional characteristics, including efficient colonic fermentability [6,7]. Moreover, citrus fruits have better quality than other sources of dietary fibre due to the presence of bioactive compounds, such as phenolic compounds, with antioxidant and antimicrobial properties. These bioactive compounds may confer higher health-promoting effects than the dietary fibre itself [4,8,9]. Dietary fibre may exhibit antioxidant effects due to the presence of phenolic compounds attached through hydrogen bonds to the polysaccharides, known as bound phenolic compounds (BP). Free phenolic compounds (FP) are not bound to the fibre matrix and can be extracted with water and organic solvents, whereas BP requires extraction by acid or alkaline hydrolysis [10]. Free phenolic compounds can be absorbed in the upper parts of the gastrointestinal tract due to their free form. In contrast, BP must be released from the fibre, a process that partially can occur during gastrointestinal digestion (depending on the preprocessing) but predominantly occurs in the colon after the dietary fibre undergoes fermentation. Consequently, dietary fibre bound to (poly)phenols might have an antioxidant effect associated with the presence of these compounds [11]. For this reason, lemon co-products exhibit remarkable antioxidant potential among citrus fruits, making them an optimal choice for dietary strategies to prevent cardiovascular diseases, among others [12].

Dietary fibres offer a multitude of advantages beyond their nutritional and bioactive value, encompassing a spectrum of functional and technological attributes. These include water-holding capacity (WHC), oil-holding capacity (OHC), swelling capacity (SWC), viscosity modulation, gel-forming ability, textural enhancement, and chelating capabilities [13]. DF consumption plays an important role in decreasing intestinal transit time and increasing stool bulk, reducing blood total and/or LDL cholesterol levels and consequently reducing the risk factors for cardiovascular diseases, reducing post-prandial blood glucose and/or insulin levels and consequently managing diabetes by improving insulin sensitivity, decreasing body weight gain, and being fermentable by colonic microflora with all the benefits of positive gut microbiota modulation [14,15,16]. DF intake in industrialised nations is currently estimated to be less than 25 g per person/day. However, recommendations from the European Food Safety Authority (EFSA) and the World Health Organization (WHO) on adequate dietary fibre intake differ worldwide and by age group, but 25–30 g or more per day is widely recommended for adults [17,18]. Recommended intakes were defined as those necessary for maintaining normal laxation and cardiovascular health. However, emerging evidence points to benefits extending well beyond these by modulation of the gut microbiota [19]. The insufficient intake of DF in the population’s diet is often related to various health disorders such as colon and cardiovascular diseases, obesity, and cancer [20]. This trend has prompted research into incorporating natural fibres into food products, including processed meats, to improve their nutritional value while maintaining their sensory appeal. Among the diverse sources of fibre available, those extracted from citrus fruits stand out most due to their functional, technological, and bioactive properties [21,22,23].

Meat is an excellent source of high-quality protein, offering a well balanced ration of essential amino acids and having a high biological value. Additionally, it is an important source of micronutrients such as selenium, iron, magnesium, potassium, and sodium. Despite its nutritional benefits, meat has a notable drawback as it is deficient in dietary fibre [24]. The era of globalisation, combined with rapid urbanisation, has significantly influenced consumer behaviour and demand for many convenient processed meat products [25,26]. However, meat products lack the minimum contents of DF to meet the recommended daily fibre intake. *Mortadella*, a cooked-cured sausage, is often associated with high fat content and an unfavourable nutritional image. To address these concerns, incorporating potentially functional ingredients into *mortadella* formulations, such as lemon dietary fibre (LDF), emerges as a promising strategy to develop a healthier meat product that not only improves its nutritional profile but also aligns with evolving consumer demands for healthier food options. Additionally, the addition of DF can contribute to reducing fat and nitrite residual levels, substituting commercial preservatives, creating new textures, and improving stability during processing and storage, thereby increasing shelf-life [27,28,29,30,31]. According to the European Union (EU) regulation (EC) No 1924/2006, a claim that a product is “high in fibre” or “source of fibre” can be made when the product contains at least 6 g/100 g or 3 g/100 g fibre, respectively. These guidelines provide a framework for developing functional foods and represent an important benchmark to meet the expectations of consumers who are increasingly focused on the health and nutritional quality of their food choices.

Commercial citrus fibres have been noted for their water-binding capacity in low-fat frankfurters [31] and as a fat replacer in low-fat beef hamburgers [30]. Additionally, the incorporation of lemon albedo recovered from commercial lemons into bologna sausages has been studied for its fibre content (22–30%) [32]. Furthermore, the incorporation of fruit-derived dietary fibre powders into meat products has gained increasing attention as a strategy to enhance both nutritional and functional properties, while helping to balance protein and fibre content [33,34]. Insoluble dietary fibres recovered from fruits, such as kiwi pomace, have been studied and incorporated into meatballs, showing improvements in swelling capacity as well as water, oil, and fat holding capacities. Additionally, meatballs reformulated with 3% kiwi pomace demonstrated high overall acceptability, making them a suitable option for consumers [35].

In contrast, our research is innovative because it has developed a lemon dietary fibre-based powder (LDF) that boasts a notably higher fibre. Unlike previous studies that used commercial fibre/lemons, our research recovers natural fibre from lemon co-products, promoting sustainability and resource efficiency. With a focus on the development of innovative, clean-label, and sustainable food products, this research aims to develop an LDF from lemon co-products, evaluating the chemical, physicochemical, tecno-functional, and food preservation properties of the obtained powder. Furthermore, this study aims to explore the use of LDF in *mortadella* formulations, assessing the effects on the physicochemical, technological, and sensory properties of the product. The fibre concentrations used in the formulations were established following the regulatory claims of 3% and 6%, allowing a comparative analysis between products classified as “source of fibre” and “high in fibre”. In this way, the goal is to contribute to the development of innovative meat products that meet the growing demand for healthier, more nutritious foods without compromising the quality and taste that consumers expect.

## 2. Materials and Methods 

### 2.1. Chemicals and Reagents

Acetonitrile and methanol (HPLC-gradient grade) were purchased from VWR Chemicals (Rosny-sous-Bois-cedex, France). Ethanol 96% was purchased from LabChem (Santo Antão do Tojal, Portugal). Hydrogen peroxide 33% (*w*/*v*), nitric acid 69% (*v*/*v*) (analytical grade), trichloroacetic acid, thiobarbituric acid, zinc acetate 2-hydrate, and potassium hexacyanoferrate (II) 3-hydrate for analysis were purchased from PanReac AppliChem (Darmstadt, Germany). Griess-Ilosvays nitrite reagent for microbiology (contains acetic acid) was purchased from Merck-Millipore (Darmstadt, Germany). Folin–Ciocalteu’s reagent and hydrochloric acid 37% (*v*/*v*) (HCl) were purchased from Merck (Algés, Portugal). The 2-azinobis-3-ethylbenzothiazoline-6-sulphonic acid (ABTS), 2,2′-azo-bis-(2-methylpropionamidine)-dihydrochloride (AAPH), 2,2-diphenyl-1-picrylhydrazyl (DPPH), fluorescein, gallic acid, sodium carbonate (Na_2_CO_3_), trifluoroacetic acid (TFA), and trolox (97%) were purchased from Sigma-Aldrich (Sintra, Portugal). The standards (HPLC grade; ≥95% purity) vanillin, eriocitrin, p-coumaric acid, ferulic acid, apigenin-7-O-glucoside, hesperidin, rutin, luteolin-7-O-glucoside, and hesperetin were purchased from Extrasynthese (Genay, France).

### 2.2. Lemon Dietary Fibre (LDF)-Based Powder Preparation

The lemon co-products (*Eureka* cultivar) were collected from Frutas Tereso company (Algarve, Portugal). The lemons used for fibre extraction were cultivated using pest control substances, and organic fertilisers approved for organic farming were also applied. The fertilisation includes the application of bludiamond classic 5-3-10 and bludiamond azo N-32. Foliar fertilisation is carried out using a combination of products, including nutriman N-24, sergomil, urea 46% yara, sergomax L90, feedser, verdezin extra, servalac 10-5-30, bioalgae, power stop, and red KSC, all aimed at enhancing nutrient uptake and improving plant vigour. In addition, phytosanitary treatments are performed to protect crops against pests and diseases, using products such as nutriman N-24, peters professional 20-20-20, ninja zeon, verdezin extra, urea 46% yara, repulser +3, and envidor. 

The trees were grown on clayey-sandy soil and irrigated using a drip (drop-by-drop) irrigation system. The lemons were produced under an integrated production system, with non-mobilised soil and grassing between tree rows (inter-row grass cover).

**Figure 1:**
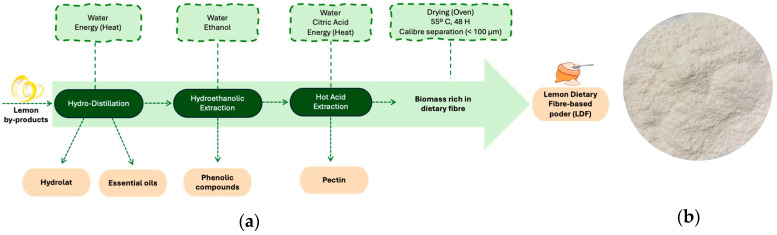
(**a**) Process flow chart: recovery of lemon dietary fibre (LDF)-based powder; (**b**) final LDF-based powder (*Eureka* cultivar).

After the integrative extraction of the lemon co-products, bioactive fractions, hydrolat, essential oils (EOs), phenolic compounds (PCs), and pectin, a dietary fibre-rich biomass was obtained and carefully dried in an oven at 55 °C for 48 h (Figure 1a). Following the drying phase, the biomass underwent grinding and granulometric separation, producing a finely powdered substance with a particle size below 100 µm. This process resulted in a lemon dietary fibre-based powder, as illustrated in Figure 1b.

### 2.3. Chemical Composition Analysis

#### 2.3.1. Proximate Composition

Dry matter (DM), moisture, ash, protein, fat, carbohydrates, and dietary fibre were determined in triplicate according to standard procedures using the AOAC methodology [36]. The results were expressed as g/100 g of product (dry weight).

#### 2.3.2. Mineral Composition

Minerals were quantified after mineralisation of the lyophilised samples (0.5 g) with 67% nitric acid and 33% hydrogen peroxide by a microwave system using Inductively Coupled Plasma Mass Spectrometry (ICPMS-2030-Shimadzu, Kyoto, Japan). The final value was the average of three reads. Mineral content was expressed in mg/100 g of product.

#### 2.3.3. Cellulose, Hemicellulose, and Lignin

For the determination of cellulose (as glucose), hemicellulose (as arabinose, mannose, galactose, and xylose), and lignin (soluble and insoluble) in lemon dietary fibre-based powder, the methodology of Ribeiro et al. (2020) was followed [37]. LDF samples were submitted to a two-step sequential acid hydrolysis, and further determination/quantification of structural carbohydrates was achieved using High-Performance Liquid Chromatography (HPLC) [37]. The insoluble lignin content was calculated gravimetrically after hydrolysis residue filtration, and soluble lignin was estimated by ultraviolet (UV) spectrophotometry at 340 nm [38]. The results were expressed in g/100 g DW.

### 2.4. Physicochemical Properties

#### 2.4.1. pH and Water Activity (a_w_)

The pH was measured using a calibrated pH metre GLP21 (Crison, Barcelona, Spain) on a 10% (*w*/*v*) aqueous suspension for 2 min. The water activity (a_w_) was determined using a LabSwift-aw Novasina Hygrometer (Lachen, Switzerland) at 25 °C.

#### 2.4.2. Colour

The colour was measured using a CM-700 Minolta spectrophotocolourimeter (Minolta, Osaka, Japan) in CIELAB mode under CIE Standard Illuminant D65 and an observation angle of 10°. The lemon dietary fibre-based powder was placed in a Petri dish for colour measurements, and six readings were performed at room temperature (25 °C). The following CIELAB coordinates were determined: lightness (L*), redness (a* ± red/green), and yellowness (b* ± yellow/blue). From these coordinates, psychophysical magnitudes chrome (C*) and hue (h°) were calculated using Equations (1) and (2), respectively. Colour measurements were made according to the American Meat Science Association (AMSA) guidelines for colour assessment [39].

(1) $$C^{*}=\sqrt{a^{*2}+b^{*2}}$$

*h°* = arctg (b*/a*) (2)

### 2.5. Techno-Functional Properties

The water-holding capacity (WHC), oil-holding capacity (OHC), and swelling capacity (SWC) were determined according to the methodology described by Fernández-López et al., 2015 [21]. The analysis was performed in triplicate.

#### 2.5.1. Gelling Capacity and Precipitate in the Oily Phase

Gelling capacity (GC) and precipitate in the oily phase (POP) are defined as the capacity of the sample to absorb fat and water in a matrix, forming a gel. The analysis was made in triplicate. Distilled water and sunflower oil (20 g of each one) were mixed, heated, and centrifugated. Two phases, the gel phase and precipitate in the oily phase, were identified in the tubes, and the relation between these two phases concerning the total volume was calculated. The GC and POP were calculated according to Equations (3) and (4), respectively.

(3) $$\mathrm{GC}=\frac{Gel\;layer\;volume}{Total\;sample\;volume}\times 100$$

(4) $$\mathrm{POP}=\frac{Precipitate\;layer\;volume}{Total\;sample\;volume}\times 100$$

#### 2.5.2. Emulsifying Activity and Emulsion Stability

Emulsifying activity (EA) and emulsion stability (ES) were carried out following the methods described by Chau and Huang (2003) [40]. The analysis was made in triplicate. The EA and ES results were calculated according to Equations (5) and (6), respectively.

(5) $$\mathrm{EA}=\frac{Emulsified\;layer\;volume}{Total\;sample\;volume}\times 100$$

(6) $$\mathrm{ES}=\frac{Remaining\;emulsified\;layer\;volume}{Original\;emulsion\;volume}\times 100$$

### 2.6. Structural Characterisation

#### 2.6.1. Fourier Transform Infrared Spectroscopy (FT-IR)

Infrared spectra were obtained by analysing the sample with the Spectrum 100 FT-IR Spectrometer (Perkin Elmer, Waltham, MA, USA), with an attachment of total attenuated internal reflection (ATR) in the spectral region between 500 cm^−1^ and 4000 cm^−1^. The resolution used was 2.0 cm^−1^, and each spectrum is the average of 100 scans in transmittance (%T) mode.

#### 2.6.2. Scanning Electron Microscopy (SEM)

The morphology of the LDF samples was evaluated using a Phenom XL G2 (Thermo Fischer Scientific, Waltham, MA, USA) scanning electron microscope (SEM). Sample powder was placed over double-sided adhesive carbon tape (NEM tape; Nisshin, Nisshin-shi, Japan), which covered the observation pins and were coated with gold/palladium on a sputter coater (Polaron, Bretzfeld, Germany). An acceleration voltage of 20 kV in high-vacuum mode was used; all images were obtained using the secondary electron detector and are representative of the morphology of the sample.

### 2.7. Bioactive Characterisation

#### 2.7.1. Free and Bound Phenolic Compound Extraction

Free phenolic (FP) compound extracts from LDF were prepared as described by Tinh et al. (2021) with some modifications [41]. LDF was extracted with water (H_2_O) and compared with methanol (MeOH). It was then homogenised using an Ultra-Turrax digital apparatus (IKA T18, Wilmington, NC, USA) at 20,000 rpm for 1 min. Then, the mixture was sonicated using an ultrasonic bath for 60 min and left under continuous stirring (200 rpm) at room temperature (25 °C) overnight. Lastly, the mixture was centrifuged at 10,000 rpm at 4 °C for 15 min, and the supernatants were collected and filtered using a 0.45 µm filter (Orange Scientific, Braine-l’Alleud, Belgium).

Bound phenolic (BP) compound extracts from LDF were determined with alkaline and acid hydrolyses according to the method described by Ribeiro et al. (2020) with some modifications [37]. Briefly, the free phenolics solid residue was hydrolysed for 4 h with 20 mL of 4 M NaOH in an orbital shaker at 250 rpm. After that, the mixture was acidified to pH 2.0 using HCl and centrifuged (8000 rpm, 15 min at 4 °C); the supernatant was extracted 3 times with ethyl acetate (15 mL). This extract was Alkaline Bound Phenolics (ALK-BP). The pellet obtained after alkaline hydrolyses was submitted to an acid hydrolyse with 5 mL of 2M HCl for 1 h at 85 °C. After that, the mixture was acidified to pH 2.0 using NaOH and centrifuged (8000 rpm, 15 min at 4 °C); the supernatant was extracted 3 times with ethyl acetate (15 mL). This extract was Acid Bound Phenolics (ACD-BP). The ethyl acetate of the extracts was evaporated to dryness using a rotary vacuum evaporator at 40 °C. The resulting residue was subsequently dissolved in methanol or water to a final volume of 5 mL. The extracts obtained were stored at −80 °C until analysis.

#### 2.7.2. Total Phenolic Content (TPC)

The TPC of the LDF extracts (in MeOH and H_2_O) was determined by the Folin–Ciocalteu method followed by Vilas-boas et al. method (2020) with some modifications [42]. Gallic acid was used as a standard for the calibration curve (0.025–0.200 mg/mL), and the results were expressed as milligrams equivalent of gallic acid per gram of dry weight (mg GAE/g DW). The analyses were performed in triplicate.

#### 2.7.3. Phenolic Compound Quantification by HPLC-DAD

The quantitative profiling of phenolic compounds (PCs) in LDF extract was conducted using a Waters Alliance e2695 separation module system interfaced with a photodiode array UV/Vis detector 2998 (PDA 190–600 nm; Waters, Mildford, MA, USA). The separation took place in a reversed-phase C18 column (ZORBAX Eclipse XDB-C18, 80 A°; 4.6 × 250 mm; 5 µm; Agilent, Santa Clara, CA, USA) at 25 °C. The mobile phase, the gradient elution, the injection volume, and the flow rate were prepared according to [42]. Data acquisition and analysis were carried out using Software Empower 3. Detection was performed at 280, 320, and 350 nm, and phenolic compound identification was achieved by comparing the retention time and absorbance spectra with pure standards. Quantification was conducted using calibration curve interpolation, and the results were expressed as milligrams per 100 g of dry weight (mg/100 g DW). The analyses were performed in triplicate.

#### 2.7.4. Antioxidant Capacity

##### ABTS Assay

The free radical scavenging capacity of the LDF extract, described in Section 2.6.1, was evaluated using the ABTS (2,2′-azino-bis-(3-ethylbenzothiazoline-6-sulfonic acid) radical cation decolourisation assay [43]. The results were expressed as micromoles of Trolox equivalent per gram of extract (µmol TE/g). The analyses were performed in triplicate.

##### DPPH Assay

The electron donation capacity of the extracts was measured by bleaching the purple-coloured solution of 1,1-diphenyl-2-picrylhydrazyl radical (DPPH) according to the method of Bondet et al. (1997) with some modifications [42,44]. Trolox was used as a standard to prepare a calibration curve (0.025–0.175 µmol/mL). The results were expressed as micromoles of Trolox equivalent per millilitre of extract (µmol TE/mL). The analyses were performed in triplicate.

##### Oxygen Radical Absorbance Capacity Assay (ORAC)

The ORAC assay, which belongs to the hydrogen atom transfer (HAT)-based assay family, gives a measure of species competing for peroxyl radicals and was carried out following the method established by Dávalos et al. (2004) [45]. Each plate experiment also included a calibration curve (1–8 µM of Trolox). The excitation wavelength was set at 485 nm and the emission wavelength at 528 nm [46]. The software used was the Multidetection plate reader (Synergy H1, Winooski, VT, USA) controlled by the Gen5 Biotek software version 3.04. All analyses are conducted in triplicate. Antioxidant curves (fluorescence versus time) were normalised to the blank curve corresponding to the same assay by multiplying the original data by fluorescenceblank, t = 0/fluorescencesample, t = 0. Employing the normalised curves, the area under the fluorescence decay curve (AUC) was calculated according to Equation (7).

(7) $$AUC=\sum_{i=1}^{i=n-1\;}(\frac{f_{i}\;{+f}_{i+1}}{2})*(t_{i+1}-t_{i})$$

where $f_{i}$ is the fluorescence at reading I and $t_{i}$ is the time (minutes) at reading *i*.

#### 2.7.5. Antibacterial Activity

##### Bacterial Culture

The following bacterial strains were used: *Escherichia coli* (ATCC (American Type Culture Collection) 25922) and *Salmonella enterica* (ATCC 13076), *Bacillus cereus* (NCTC (National Collection of Type Cultures) 2599), and *Staphylococcus aureus* (ATCC 25923). These bacteria were cultured in Mueller–Hinton broth (MHB) and incubated at 37 °C overnight. After the incubation, the inoculum concentration for testing was ca. 10^8^ CFU/mL.

##### Determination of Bacterial Inhibition

The bacterial inhibition of LDF was tested for 3% fibre concentration (0.349 g of LDF powder diluted in 10 mL of MHB). These samples were inoculated with bacteria at 10^6^ CFU/mL. The inoculated 24-well plates (24-well plates; Orange Scientific 4430300N) were placed in an orbital shaker at 40 rpm for 24 h at 37 °C overnight. After 24 h, serial dilutions (from −1 to −6) were performed, and 20 µL of each dilution was plated on Muller–Hinton Agar Petri Plates. Furthermore, the same procedure was carried out for a negative control (MHB only) and a positive control (bacteria without the LDF sample) to validate the experiment. All tests were conducted in triplicate. The results were expressed as a log reduction in viable cell counts and percentage reduction (%). The log reduction was calculated according to Equation (8).

Log reduction = log_10_ (A) − log_10_ (B) (8)

where A is the number of viable microorganisms before treatment, and B is the number of viable microorganisms after treatments.

### 2.8. Sausage Elaboration Process

*Mortadellas* were manufactured according to a traditional formula (only the meat percentage adds up to 100% while the percentage of the other ingredients is related to the meat): 70% lean pork meat and 30% pork backfat; 15% water (ice, *w*/*w*), 3% potato starch (*w*/*w*), 1.5% sodium chloride (*w*/*w*), 500 mg/kg sodium ascorbate, 300 mg/kg sodium tripolyphosphate, 150 mg/kg sodium nitrite, and 0.3% spices. This original mixture was used as a control, while lemon dietary fibre (LDF) (3% and 6%) was added to the other samples. The LDF was obtained after the extraction of (essential oils, phenolic compounds, and pectin), resulting after extraction into a biomass composed mainly of fibre. To achieve fibre incorporation rates of 3% and 6% in the *mortadellas*, 3.49 g and 6.99 g of LDF per 100 g of *mortadellas*, respectively, were added, accounting for the 85% of fibre present in the lemon dietary fibre-based powder.

**Figure 2:**
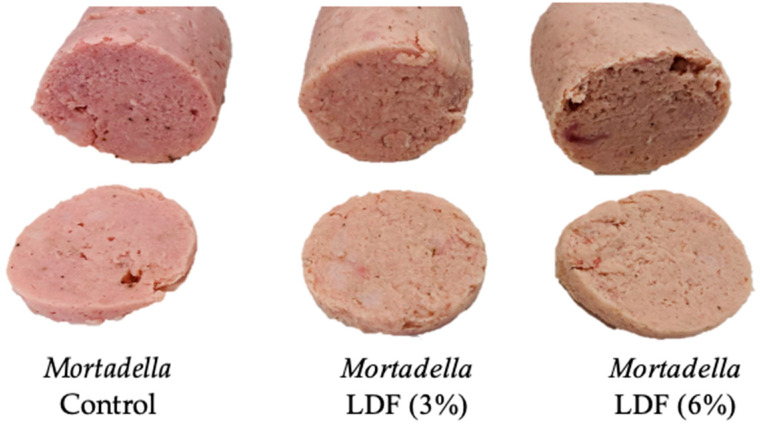
Reformulated *mortadellas* with LDF at 3 and 6%, and control.

The products were prepared in the IPOA (Innovación de Productos Alimentarios) research group pilot plant and followed industrial processing techniques. The frozen raw material of animal origin, except pork backfat, was transferred to the cutter (Tecator 1094 Homogenizer, Tekator, Höganäs, Sweden) with sodium chloride to extract salt-soluble proteins; after comminution, the other ingredients and additives were added. The pork backfat, previously cut into 10 × 10 × 10 cm cubes, was then added. After homogenisation, the mixture was stuffed into a Fibran-Pack (Fibran, Girona, Spain) artificial casing 100 × 150 mm long, clipped at both ends (Polyclip system/Niedecker, Germany), and cooked in a water bath. The sausages were kept in the bath until the coldest point (the geometric centre of each *mortadella*, corresponding to the thickest part of the product, reached 72 °C). A thermocouple probe (Omega Engineering, Inc., Stamford, CT, USA) positioned in the geometric centre of the *mortadella* was used to monitor product temperature. The sausages were immediately chilled on ice when the endpoint temperature was achieved. After reaching 20 °C, the product was transferred to the laboratory in ice-filled insulated boxes. The *mortadella* sausages (1000 g each) were stored at 4 °C until analysis for no longer than two weeks. Three replications of this elaboration process were performed on different days (Figure 2).

### 2.9. Emulsion Stability of Meat

The emulsion stability of meat batters (before cooking) was assessed by measuring the total expressible fluid (TEF) following the method of Botella-Martínez et al. (2021) with slight modifications [47]. TEF represents the proportion of fluid separated from the emulsion, serving as an indicator of stability. A higher %TEF represents reduced stability as more fluid is released, whereas lower %TEF values indicate greater emulsion stability with minimal fluid separation.

The samples were initially centrifugated at 3000 rpm for 1 min. Subsequently, they were heated in a water bath at 70 °C for 30 min and then cooled to room temperature. Next, they were centrifuged again for 3 min at 3000 rpm. The samples were left standing upside down to allow the expressible fluid (fat and water) to flow into filter paper. All determinations were conducted in triplicate for each sample. The results are expressed in grams of total fluid expelled per 100 g of sample and were calculated using the following expression (Equation (9)):

(9) $$\%TEF=\frac{Weight\;of\;tube\;with\;sample-Weight\;of\;tube\;with\;pellet}{Weight\;of\;sample}\times 100$$

### 2.10. Chemical Analysis

#### 2.10.1. Proximate Composition

Moisture, ash, lipid, and protein were determined using the corresponding AOAC methods [48]. Moisture content (g water/100 g sample) was determined by drying a 3 g sample at 105 °C to constant weight. Ash content was measured after incineration at 550 °C for 2 h (g ash/100 g sample). Lipid content (g lipids/100 g sample) was determined by weight loss after a 6-cycle extraction with petroleum ether in a Soxhlet apparatus. Protein (g protein/100 g sample) was analysed according to the Kjeldahl method, with a nitrogen to crude protein conversion factor of 6.25.

#### 2.10.2. Minerals Composition

Minerals were quantified after mineralisation of the lyophilised raw samples (0.5 g) with 67% nitric acid and 33% hydrogen peroxide using a microwave system. Analysis was performed using Inductively Coupled Plasma Mass Spectrometry (ICP-MS-2030-Shimadzu, Kyoto, Japan). Final values represent the average of three replicates, expressed in mg/100 g of DW.

### 2.11. Technological Properties

#### 2.11.1. pH and Water Activity (a_w_)

The pH of *mortadellas* was measured directly using a pH metre (Model 507, Crison, Barcelona, Spain) equipped with a Crison combination electrode probe (Cat. no. 52, Crison, Barcelona, Spain). Measurements were performed three times, changing the position of the electrode insertion. The water activity (a_w_) was assessed with a LabSwift Novasina Hygrometer (Novasina; Axair Ltd., Pfaeffikon, Switzerland) at 25 °C.

#### 2.11.2. Colour

Colour measurements were conducted using the CIELab colour space with a CM-2600 d colourimeter (Minolta Camera Co., Osaka, Japan) using illuminant D65, an observer angle of 10°, SCI mode, 11 mm aperture for illumination, and 8 mm for measurement. The samples were placed in Petri dishes for measurement, and the following colour parameters were recorded: lightness (L*), redness (a* ± red-green), and yellowness (b* ± yellow-blue). From these coordinates, hue (h* = tan − 1 b*/a*) and chroma (C* = (a*2 + b*2) 1/2) were calculated. Nine determinations per sample were carried out [(three measures on the same inner face of three slices (2 cm height)].

#### 2.11.3. Textural Properties

Texture profile analysis (TPA) was conducted using a TA-XT2 Texture analyser (Stable Micro Systems, Surrey, England). *Mortadella* samples were removed from the casing, cut into cubes (1 × 1 × 1 cm), and subjected to a 2-cycle compression test. All texture analyses were performed on chilled (4 °C) samples. Each sample was subjected to a two-cycle compression to 75% of its original height with a speed of 1 mm/s, and at 20–25 °C, the corresponding force–time deformation curves were obtained. The texture profile, evaluated according to Bourne (1978) [49], included hardness (N), springiness (mm), cohesiveness, and chewiness (N × mm). Each sample was analysed in six replicates.

### 2.12. Residual Nitrite Level

The residual nitrite level (mg NaNO_2_/kg sample) was determined according to standards ISO/DIS 2918.26 [50].

### 2.13. Lipid Oxidation

Lipid oxidation was evaluated in triplicate using the 2-thiobarbituric acid (TBA) test following the recommendations of Buege and Aust (1978) [51]. TBAR values were calculated from a malonaldehyde (MDA) standard curve and expressed as mg MDA/kg sample. Briefly, the extracts were prepared by mixing the sample with TBA and trichloroacetic acid solutions, followed by heating and centrifugation. The absorbance of the resulting supernatant was then measured at 532 nm.

### 2.14. Sensory Evaluation

A sensory panel of 45 untrained participants (20 males, 25 females) aged 18–60 years was recruited from the staff and students at Miguel Hernández University to evaluate the three *mortadella* formulations. The protocols for sensory analysis were accepted by the Project Evaluation Office of the Miguel Hernández University (OEP, UMH, Elche, Alicante, Spain). This analysis was operated under white, fluorescent lights in individual booths. Three pieces (ca. 1.0 cm^2^), one from each bath, were cut from the *mortadellas* and served at room temperature. Unsalted crackers and mineral water (room temperature) were provided to clean the palate between samples. A 9-point hedonic scale (1: dislike extremely and 9: like extremely) was used to evaluate the following attributes: global appearance, colour appearance, general quality, hardness, homogeneity, general flavour, acid taste, and bitter taste.

### 2.15. Statistical Analysis

Conventional statistical methods were used to calculate means and standard deviations and are shown in corresponding tables and figures. Data were evaluated by one-way analysis of variance (ANOVA), and if statistically significant differences were found, a Tukey post hoc test was performed at a 5% significance level (*p* < 0.05). For the *mortadella* characterisation, three samples were obtained from each of the three formulations during each elaboration process (3). ANOVA was applied for each parameter with one-factor treatment and three levels (control, LDF (3%), and LDF (6%)). The statistical analyses were made using GraphPad Prism Software (version 8).

## 3. Results and Discussion

### 3.1. LDF Analysis

#### 3.1.1. Proximate Composition

The proximate composition analysis of lemon dietary fibre-based powder (LDF) recovered from lemon co-products (*Eureka* variety) is outlined in Table 1. LDF is primarily composed of total dietary fibre (TDF), which accounts for approximately 85.8% of its composition. Within the TDF fraction, insoluble dietary fibre (IDF) makes up 52.6%, while soluble fibre (SDF) represents 33.2%. Additionally, LDF is predominantly composed of carbohydrates (90.5%), protein (4.6%), and ash (1.2%). The composition analysis of LDF regarding structural carbohydrates was determined by HPLC and revealed the presence of cellulose (9.6%), hemicellulose (6.8%), soluble lignin (9.9%), and insoluble lignin (5.6%).

Lemon co-products typically contain high levels of dietary fibre, which vary depending on factors such as cultivar, variety, and origin. Generally, they have higher concentrations of insoluble fibre compared to soluble fibre. For instance, lemons with minimal albedo (the white part of the peel) inherently have a lower fibre content. According to research by Nempeque et al. (2021), dried lemon (*Citrus latifolia*) peels contained 92.86% of dietary fibre [52]. Wang et al. (2015) assessed the fibre content of dried lemon peels (*Citrus limon* (L.) Burm.f.) sourced from commercial orchards in China [53]. These peels contained 64.07% TDF, with IDF accounting for 50.32% and SDF representing 12.89%. Similarly, a research group reported comparable values when producing dietary fibre from lemon peel waste, with SDF and IDF representing approximately 19.55% and 80.56%, respectively [54]. A recent study by Xiao et al. (2024) evaluated the dietary fibre content of lemon peels (sourced from China) after extracting lemon oil and juice [55]. The resulting powder contained 73.66% TDF, comprising 22.05% SDF and 51.58% ISF. Additionally, the composition included 26.70% cellulose, 12.43% hemicellulose, and 5.39% lignin.

#### 3.1.2. Mineral Composition

The mineral quantitative profile of lemon dietary fibre-based powder is shown in Table 2. The most abundant mineral was calcium (Ca), 425.96 ± 4.54 mg/100 g, followed in descending order by phosphorus (P), 184.60 ± 7.27 mg/100 g, and potassium (K), 77.92 ± 6.53 mg/100 g. The mineral profile observed in the LDF may be influenced by the agro-technical practices employed during cultivation. The use of organic fertilisers, clayey-sandy soil, and drip irrigation can influence and affect the nutritional composition of fruits and promote mineral uptake. Therefore, it is plausible that the growing conditions in this study contributed to the mineral content of the lemon fibre [56,57]. Minerals can be divided into macro-minerals, such as potassium, calcium, and magnesium, and micro-minerals, such as iron, zinc, copper, and manganese. Macro-minerals play a vital role and are required by the human body in large quantities [58]. Essential minerals, which the body cannot produce, must be obtained from the diet. Poor-quality diets can result in deficiencies, with calcium, phosphorus, and potassium being a significant concern [59]. Furthermore, WHO recommends a daily intake of 1000 mg of calcium daily [60], while EFSA advises a phosphorus consumption of 700 mg/per day for young adults [61].

Minerals have been documented in the literature as having important physiological roles. Czech et al. (2020) reported that lemon peel from the *Citrus limon*, Interdonato cultivar (Turkey), showed more macro- and micro-minerals than lemon pulp and the whole fruit [62]. The main macro-minerals detected in lemon peel were potassium (127.00 mg/100 g), calcium (31.80 mg/100 g), and phosphorus (23.90 mg/100 g). Regarding micro-minerals, lemon peel had substantially lower quantities, with the principal constituents being iron (0.34 mg/100 g) and zinc (0.28 mg/100 g). Trace element levels in fruit may be influenced by various factors, including the soil’s mineral composition, the quality of irrigation water, weather conditions, and agricultural practices such as fertiliser usage and fruit variety. However, detailed information on the minerals present in lemon co-products remains limited. Furthermore, Lario et al. (2004) formulated a fibre-rich powder but did not analyse its mineral content [12]. Therefore, our study is particularly relevant, as we have successfully developed a powder that is not only rich in fibre but also contains essential minerals crucial to promote health and well-being.

### 3.2. Physicochemical Properties

#### pH, a_w_, and Colour

Table 3 shows the physicochemical properties (pH, a_w_, and CIELAB colour coordinates) of the LDF. The pH is one of the most critical parameters affecting their processing and storage quality. The LDF exhibited an acidic pH of 3.27. This value is consistent with previous studies. For instance, Fernández-López et al. (2015) [21] reported pH values for lemon fibres ranging from 3.84 to 4.70, while Lario et al. (2004) [12] observed pH values between 3.83 and 3.98 for dried lemon fibre. Water activity (a_w_) indicates available water for biological functions. Lowering a_w_ reduces microbial growth. The limit value of aw for microbial growth is around 0.6, and below this, food is microbiologically safe [63]. The water activity in LDF is 0.424. Bakshi and Ananthanarayan (2022) [64] reported a similar aw value for lemon peel powder (0.45). In contrast, other studies reported even lower aw values (0.13–0.21) for lemon fibres, likely due to their lower moisture content [12].

Colour is one of the most important quality parameters in food ingredients/products. L* represents lightness from black to white on a scale of 0 to 100 and correlates with the moisture content; L* values increase as moisture content increases. L* value in LDF was 77.91, which follows other authors’ reports (64.91–78.53) [12]. The a* values represent (red (+)/green (−)), and the b* values indicate (yellow (+)/blue (−)) components that define the colour chromaticity. LDF showed higher yellow components (b* = 13.59) than red components (a* = 0.69), which is characteristic of lemon fruits and their co-products. C* (Chroma), which means the intensity or purity of the colour. A higher C* value suggests a more vibrant or saturated colour, while a lower value indicates a more muted or desaturated colour. Therefore, the LDF (C* = 13.74) indicates how far the colour is from being saturated. On the other hand, h° denotes the hue angle, representing the specific colour direction on the CIELAB colour wheel. In this case, 86.89° in LDF is close to the yellow-green area.

### 3.3. Structural Characterisation Analysis

#### 3.3.1. FT-IR Spectra

**Figure 3:**
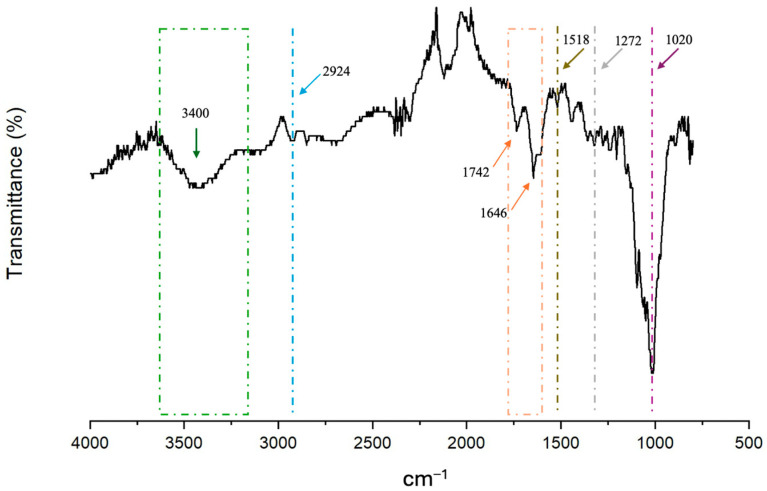
FT-IR spectra in the 4000–500 cm^−1^ region of the lemon dietary fibre-based powder.

Fourier Transform Infrared Spectroscopy (FT-IR) is a widely used analytical technique for detecting and quantifying chemical bonding species and functional groups within a sample. It serves as a valuable tool for evaluating proteins, fibres, and other substances. The FT-IR spectrum of LDF is presented in Figure 3. According to the infrared spectra analysis, LDF had a characteristic peak near 3400 cm^−1^, due to the stretching vibration of O–H, which is the characteristic band of cellulose. The absorption peak at 2924 cm^−1^ represents the stretching vibration peak of aliphatic saturated C–H in cellulose and hemicellulose [65]. Absorption peaks at 1742 cm^−1^ and 1646 cm^−1^ are attributed to the C=O stretching vibrations. In addition, the characteristic bending or tensile vibration peak of lignin is at 1518 and 1272 cm^−1^ [66]. The characteristic peak near 1020 cm^−1^ was attributed to the stretching vibration of the C-O-C glycosidic bond and C-O-H side group, which indicates the existence of a pyran ring. This peak is a strong indicator of the polysaccharide content in plant-based fibres.

#### 3.3.2. SEM

**Figure 4:**
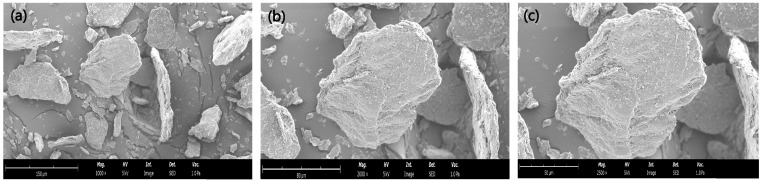
Scanning electron microscopy images (SEM) of lemon dietary fibre (LDF). (**a**) LDF: 1000× magnification and scale bar of 150 μm; (**b**) LDF:2000× magnification and scale bar of 80 μm; (**c**) LDF: 2500× magnification and scale bar of 50 μm.

The microscopic surface morphology of lemon dietary fibre-based powder was analysed by scanning electron microscope (SEM) (Figure 4). The microstructural analysis of LDF revealed important structural characteristics that directly influence its physicochemical and functional properties in food applications. At a magnification of 1000 times, the particles exhibited distinct sizes, being fragmented and with irregular shapes, ranging from small fragments to larger structures, which indicates a heterogeneous distribution. This variability can be attributed to the composition of the fibre but especially to the mechanical processing, such as milling, which typically produces particles of differing dimensions. At magnifications of 2000 and 2500 times, the overall morphology appeared flaky. The microstructure of citrus fibre plays a critical role in determining its physicochemical properties, such as water-holding capacity and cation exchange capacity. Citrus fibres with a large surface area offer more binding sites for water molecules through hydrogen bonding and/or dipole interactions. This property enhances their functionality, making them valuable as texturisers, stabilisers, and moisture retainers in food formulations [67].

### 3.4. Techno-Functional Properties

**Figure 5:**
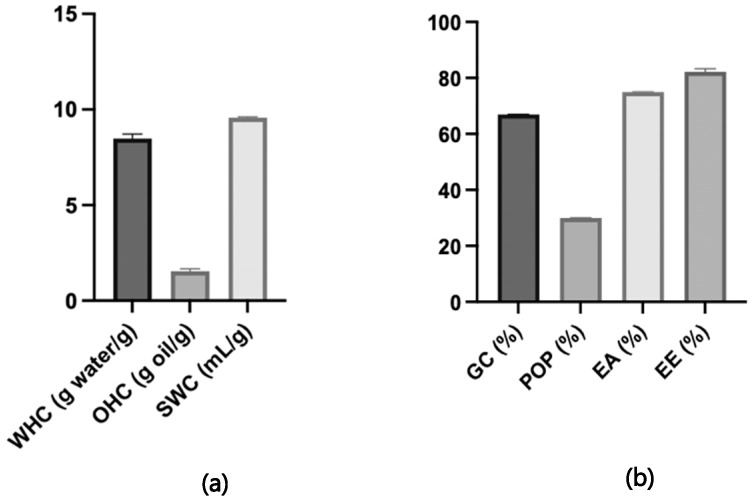
Techno-functional properties: (**a**) (WHC, water-holding capacity (g water/g); OHC, oil-holding capacity (g oil/g); SWC, swelling capacity (mL/g)); (**b**) (GC, gelling capacity (%); POP, precipitate in oily phase (%); EA, emulsion ability (%); EE, emulsion stability (%)) of lemon dietary fibre-based powder. Data are presented as mean ± SD, where n = 3.

Figure 5 shows the techno-functional properties of the lemon dietary fibre-based powder (LDF). The hydration properties, evaluated as their water-holding capacity (WHC) and swelling capacity (SWC), are beneficial in determining their optimal usage levels in foods as they contribute to desirable texture properties. The WHC of fibre relies on its processing, chemical composition, and physical structure, particularly influenced by its soluble dietary fibre content. Dry milling alters the physical structure of lemon fibre, compacting it and reducing its water-holding capacity by increasing fibre density through broken pores. However, the prominent WHC (8.49 ± 0.24 g water/g) and SWC (9.57 ± 0.04 mL/g) of LDF indicate its potential as a functional ingredient in food applications. It can help to mitigate syneresis, modify the texture and viscosity, and contribute to the calorie reduction in foods. Oil-holding capacity (OHC) is another important technical property of dietary fibre, which plays an important role in preventing fat loss during food processing, and LDF showed an OHC of 1.55 ± 0.13 g oil/g. In addition, LDF showed a gelling capacity (GC) of 67%, precipitation in the oil phase (POP) of 30%, emulsifying ability (EA) of 75%, and emulsion stability (EE) of 82%. These properties suggest that a significant portion of LDF interacts with the oil phase, enhancing emulsion stability. As a result, LDF is highly effective in forming and maintaining stable emulsions, making it a valuable ingredient for improving texture in meat products. Its ability to retain moisture and stabilise oil–water mixtures contributes to a more consistent and desirable texture in processed meats. The existing research has been mainly focused on evaluating the WHC, OHC, and SWC. A study conducted by Núñez-Gómez et al. (2024) [68] reported a SWC of 9.9 mL/g, a WHC of 1.1 g water/g, and an OHC of 9.3 g oil/g in lemon peels. These results differ from our findings, as their study demonstrated a higher fat absorption capacity and a lower water retention capacity in lemon peels. In contrast, Huang et al. (2021) [69] observed functional property values in untreated citrus peels that are more in line with our results, reporting an SWC of 8 mL/g, a WHC of 8 g/g, and an OHC of 2 g/g. Similarly, a recent study by Xiao et al. (2024) [55] also presented values comparable to our findings, further supporting the consistency across different studies. These previous studies have focused on evaluating the techno-functional properties of citrus peels. The novelty of this research lies in being the first to evaluate these properties in lemon dietary fibre obtained through a biorefinery extraction approach.

### 3.5. Bioactive Properties

#### 3.5.1. Total Phenolic Content

**Figure 6:**
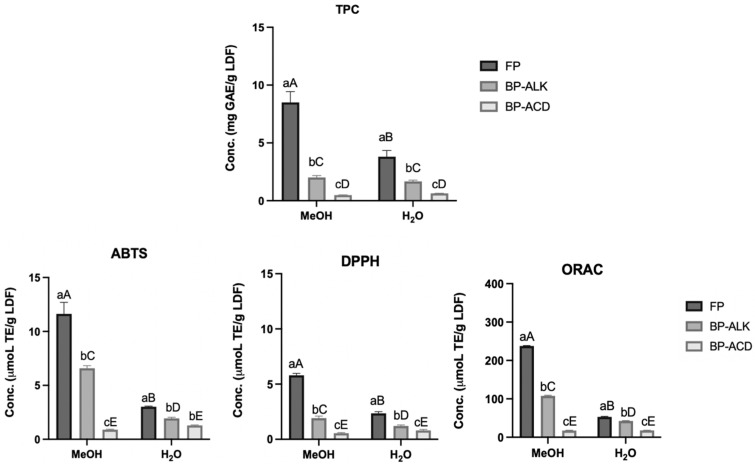
Total phenolic content (TPC) and antioxidant activity (ABTS, DPPH, and ORAC) of LDF in free phenolic extracts (FP), bound phenolic extracts obtained by alkaline hydrolysis (BP-ALK), and by acid hydrolysis (BP-ACD). Different lowercase letters indicate significant differences between extraction types (FP, BP-ALK, and BP-ACD), while different uppercase letters indicate significant differences between solvents (MeOH and H_2_O) (*p* < 0.05), as determined by Tukey’s Multiple Range Test. Data are presented as mean ± SD, where n = 3.

The total phenolic content (TPC) evaluated and compared across three types of extracts, free phenolic extracts (FP), bound phenolic extracts obtained via alkaline hydrolysis (BP-ALK), and bound phenolic extracts obtained via acid hydrolysis (BP-ACD), are illustrated in Figure 6. Furthermore, the differences in TPC among these extracts were analysed using two solvents: methanol (MeOH) and water (H_2_O). The results revealed that FP extracts consistently exhibited the highest TPC levels, irrespective of the solvent used. Notably, the use of MeOH as a solvent resulted in a significantly higher TPC concentration in FP extracts compared to water (*p* < 0.05). In contrast, both BP-ALK and BP-ACD extracts displayed lower TPC levels, with the solvent type having no significant effect on their TPC concentrations (*p* > 0.05). A study conducted by Al-Qassabi et al. (2018) evaluated the TPC in imported lemon peels using six different extraction methods [70]. The study found that methanol extracts exhibited the highest TPC (7.75 mg/g), while water extracts had the lowest (5.34 mg/g). These results are consistent with ours and further support the efficacy of methanol as a solvent for extracting PCs from lemon co-products.

#### 3.5.2. Phenolic Compound Quantification by HPLC-DAD

The quantification of phenolic compounds by High-Performance Liquid Chromatography with Diode Array Detection (HPLC-DAD) in FP, BP-ALK, and BP-ACD extracts is presented in Table 4. Hesperidin and eriocitrin were identified and quantified in free phenolic extracts as the main phenolic compounds. Both compounds were found in significantly higher concentrations when methanol was used as the solvent compared to water (*p* < 0.05). For hesperidin, the concentration was 894 mg/100 g DW with MeOH, compared to 89.97 mg/100 g DW with H_2_O. Similarly, eriocitrin was measured at 146 mg/100 g DW MeOH, versus 68.85 mg/100 g DW with H_2_O. Regarding the non-extractable fractions, normally called “bound phenolic compounds”, most phenolic compounds were identified and quantified using the first alkaline hydrolysis extraction (BP-ALK). This is likely due to the fact that alkaline hydrolysis effectively breaks ester bonds that link phenolic compounds to cell wall components such as lignin and hemicellulose. As a result, these bound phenolics are released into the extractable fraction, leading to a higher recovery and improved quantification [71]. Six phenolic compounds were quantified in this fraction, ordered by concentration as follows: vanillin > apigenin-7-O-glucoside > luteololin-7-O-glucoside > p-coumaric acid > ferulic acid > rutin. However, when water was used as the extraction solvent, only three of them were detected (vanillin, p-coumaric, and ferulic acid). This highlights the significant influence of the extraction solvent on the concentrations of these phenolic compounds and their bioactivities. In the acid hydrolysis extraction (BP-ACD), only hesperetin was quantified, with concentrations ranging from 5.30 to 6.00 mg/100 g DW (*p* < 0.05).

According to Durmus and Kilic-Akyilmaz (2023), who investigated the phenolic compounds in extractable and non-extractable fractions of lemon peel, ten phenolic compounds were identified [72]. These included caffeic acid, p-coumaric acid, ferulic acid, o-coumaric acid, rutin, hesperidin, quercetin-3-O-glucoside, quercitrin, hesperetin, and apigenin. Among these, hesperetin was detected in the highest concentrations, with 219.9 mg/100 g DW in extractable phenolics. In the non-extractable phenolics, hesperetin concentrations varied between 103.4 and 423.5 mg/100 g DW. Hesperidin was the second most abundant compound, with 136.3 mg/100 g DW in extractable phenolics and concentrations ranging from 107.7 to 269.4 mg/100 g DW in non-extractable phenolics, depending on the extraction methodology. Notably, the ultrasound-enzyme-assisted extraction method yielded the highest concentrations of these non-extractable phenolics. Additionally, it is important to emphasise that our results show a lower hesperetin content compared to those reported in crude lemon peels. This reduction can be attributed to the integrative biorefinery extraction process and the applied temperatures, which caused the degradation of certain phenolic compounds, thereby explaining the lower observed values.

#### 3.5.3. Antioxidant Activity

The food industry is increasingly adopting plant-based ingredients and extracts in response to concerns about the potential adverse effects of synthetic antioxidants. Among these natural sources, lemon co-products are distinguished for their significant antioxidant properties, offering both biological and economic advantages. The antioxidant activity of the FP, BP-ALK, and BP-ACD extracts from LDF was assessed using two solvents (MeOH and H_2_O) across three different chemical assays: ABTS, DPPH, and ORAC (Figure 6). It was observed that in all antioxidant assays, FP exhibited the highest activity, followed by BP-ALK and BP-ACD. These findings align with the TPC analysis results, suggesting that the higher antioxidant activity of FP is attributed to its greater TPC. However, it is noteworthy that bound phenolic compounds, especially BP-ALK, also demonstrated significant antioxidant activity, highlighting the potential of fibre-bound phenolics as valuable contributors to antioxidant activity. Among the assays, LDF showed higher activity against ABTS radicals compared to DPPH radicals. This can be explained by the fact that ABTS is effective for both lipophilic and hydrophilic compounds, while DPPH is more selective for the former. The ORAC method, which measures antioxidant potential under conditions that mimic cellular processes due to its similarity to physiological conditions, recorded higher antioxidant activities compared to both ABTS and DPPH. Additionally, statistically significant differences were observed between the two solvents for FP and BP-ALK (*p* < 0.05), while no significant differences were detected for BP-ACD (*p* > 0.05). A study conducted by Zappia et al. (2023) evaluated the antioxidant profile of a phenolic extract from lemon by-products obtained after extracting juice and essential oils using a 50% hydroalcoholic mixture as the solvent [73]. Their findings are consistent with ours, as they also observed higher antioxidant activity in the ABTS assay (19.42 µmol TE/g) compared to the DPPH assay (8.25 µmol TE/g). Furthermore, our study is particularly innovative and significant, as it investigates the antioxidant activity within the lemon dietary fibre, following an integrated extraction process that includes essential oils, phenolic compound-rich extract, and pectin. This approach underscores the potential of LDF as a valuable source of antioxidant activity.

#### 3.5.4. Antibacterial Activity

The antibacterial activity of lemon dietary fibre is presented in Table 5. As shown in Figure 6, LDF demonstrates a high water absorption capacity. When tested at a 6% concentration, the bacterial growth medium was completely absorbed, leading to the inhibition of bacterial growth. This result is likely due to the absence of a suitable growth medium rather than the intrinsic antibacterial activity of LDF. To address this issue, the antibacterial activity of LDF was evaluated at a lower concentration (3%), where sufficient medium was available for bacterial growth. The results showed that LDF exhibited the highest antibacterial activity against Gram-positive bacteria, particularly *B. cereus*, followed by *S. aureus*, which demonstrated a 0.3 log reduction in growth (*p* < 0.05). In contrast, the antibacterial activity against Gram-negative bacteria was much lower, with *E. coli* showing a 0.1-log reduction in growth (*p* < 0.05), while *S. enterica* was not significantly affected (*p* > 0.05). These findings suggest that LDF exhibits a low antibacterial effect, with a relatively greater impact on Gram-positive bacteria. Presentato et al. (2020) reported stronger antibacterial activity using pectin derived from lemon peel waste [74]. At a concentration of 3 mg/mL, a 1.5-log unit reduction for *S. aureus* was observed compared to the control, decreasing from 8.3 log CFU/mL in the control to 6.8 log CFU/mL with lemon pectin.

### 3.6. Sausage Analysis

#### 3.6.1. Emulsion Stability

**Figure 7:**
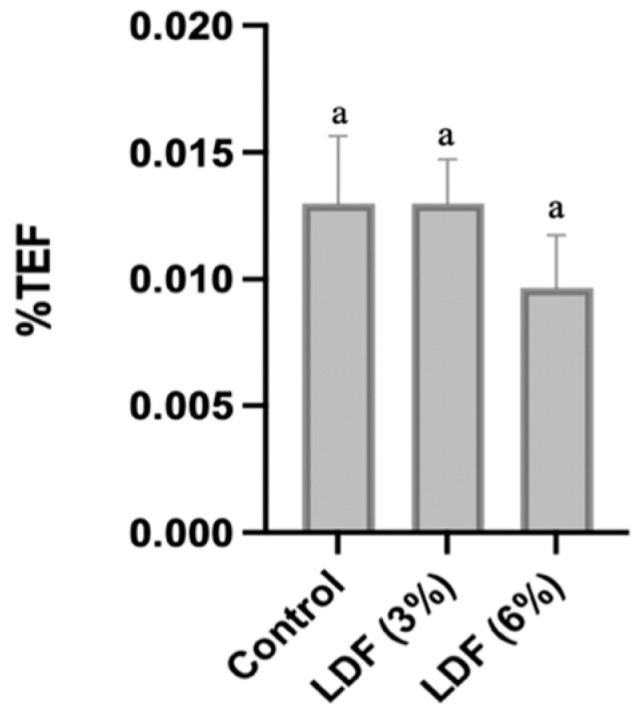
Effect of incorporation of lemon dietary fibre (3 and 6%) on emulsion stability of *mortadellas.* Results are expressed as % total expressible fluid (%TEF). For each parameter, results followed by same case letter are not significantly different according to Tukey’s HSD post hoc test (*p* > 0.05). Data are presented as mean values of replications ± SD, where n = 3.

The emulsion stability results presented in Figure 7 are expressed as the percentage of total expressible fluid (%TEF). This metric indicates the proportion of fluid that separates from the emulsion, serving as a measure of stability. A higher %TEF suggests lower stability, as more fluid is released, whereas lower %TEF values signify greater emulsion stability, with minimal fluid separation. The LDF (6%) sample exhibited a lower %TEF value (0.008%) than the other samples, indicating good emulsion stability. However, the differences in values are not significant (*p* > 0.05), probably due to the heterogeneous texture of the *mortadella*. Consequently, concentrations above 3% are required to significantly reduce fluid release. Other researchers have also assessed this parameter in meat products. Jeong and Han (2019) demonstrated that increasing the concentration of *Opuntia* fruit powder significantly reduced water and fat loss in sausages (*p* < 0.05) [75]. The group containing 10% of this powder exhibited the lowest water and fat loss, highlighting its effectiveness in improving sausage quality. Zhao et al. (2018) evaluated the effects of regenerated cellulose (0.4, 0.8, and 1.2%) on the emulsion stability of pork sausages [76]. The cellulose showed a high water-binding capacity, thereby enhancing emulsion stability.

#### 3.6.2. Chemical Composition

Table 6 shows the proximate composition of *mortadellas* formulated with 3% and 6% lemon dietary fibre. The addition of 6% lemon dietary fibre significantly (*p* < 0.05) reduced the moisture to 62.15%, compared to 66.48% in the control sample. In contrast, the levels of fat, protein, and ash did not exhibit significant differences (*p* > 0.05). Diao et al. (2024) recently investigated the effects of incorporating citrus fibres recovered from mandarin peels at concentrations of 1%, 2%, and 4% into frankfurter sausages [77]. The study revealed that significant differences in moisture and fat content were observed only in samples with 4% citrus fibre compared to the control. Ash content exhibited statistically significant differences in formulations with 2% and 4% citrus fibre relative to the control (*p* < 0.05), while protein content did not show any significant changes (*p* > 0.05).

#### 3.6.3. Mineral Composition

**Figure 8:**
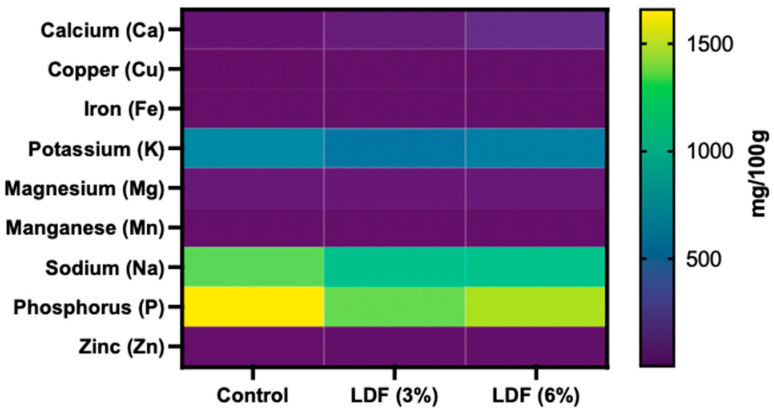
Heat map illustrating impact of incorporating lemon dietary fibre (3% and 6%) on mineral composition of *mortadellas.* Data are presented as mean values of replications ± SD, where n = 3.

The mineral composition of the *mortadella* samples is illustrated in the form of a heat map in Figure 8, which provides a detailed visualisation of each mineral’s distribution and relative abundance across the samples. The mineral composition of the *mortadella* samples included zinc (Zn), phosphorus (P), sodium (Na), manganese (Mn), magnesium (Mg), potassium (K), iron (Fe), copper (Cu), and calcium (Ca). Among these, the highest concentration was for P, followed by Na and K. Statistically significant differences were observed in the levels of P and K across all samples (*p* < 0.05). However, Na exhibited significant differences between the control sample and those containing 3% and 6% of LDF but not between the two LDF treatments (3% and 6%). This indicates that while P and K varied consistently among all samples, Na content was specifically influenced by the presence and concentration of LDF. The incorporation of LDF into *mortadellas* significantly reduced Na levels (*p* < 0.05), highlighting its potential as a viable strategy for Na reduction. This is particularly important given the critical role of Na salts as additives in food processing, where they contribute to flavour enhancement, texture improvement, and food preservation. However, excessive Na intake is a global health concern associated with cardiovascular and cerebrovascular diseases [78]. Worldwide, sodium salt intake exceeds the maximum recommended level by the WHO (2012) (salt not exceeding 5 g/day, sodium not exceeding 2 g/day) [79]. In Europe, the average sodium salt intake is about 8–12 g/day. Efforts to reduce sodium levels face several challenges, including a decline in both their textural properties and characteristic flavour. Consequently, current research has focused on achieving sodium reduction without compromising saltiness or texture quality.

This study demonstrates that lemon dietary fibre can serve as a natural, clean-label alternative for lowering sodium levels in processed meat products. This approach addresses consumer demand for healthier options without compromising product quality, making it a promising solution in the ongoing pursuit of a balanced and healthy diet. Recent research by Powell et al. (2019) evaluated the effects of replacing sodium tripolyphosphate with citrus fibre (0.5%, 0.75%, and 1%) in a bologna sausage [29]. The results showed that this replacement did not significantly affect most physical, chemical, or sensory characteristics of the product during refrigerated storage.

#### 3.6.4. Physicochemical Parameters

##### pH and Water Activity

The pH and water activity (a_w_) values for the *mortadella* samples are presented in Table 7. The results indicate that the incorporation of LDF had a significant impact on the pH of the samples (*p* < 0.05). The natural acidity of the lemon fraction LDF contributed to a reduction in pH, resulting in more acidic *mortadella* compared to the control. Among samples, LDF at 6% had the lowest pH value of 5.36, followed by LDF at 3% with a pH of 5.66, while the control sample had the highest pH at 6.02. Although in this study we did not incorporate the whole lemon peel flour, these findings are in accordance with those reported by Ibrahim et al. (2018), who incorporated 1 and 2% dried lemon peel in beef patties and observed a reduction in pH values to 5.80 and 5.71, respectively, compared to the control sample, which had a pH of 6.01 [80]. This demonstrates the acidifying effect of lemon peel, consistent with its expected acidity. In contrast, a_w_ did not show significant differences among the samples (*p* > 0.05). This indicates that the addition of LDF, irrespective of its concentration, did not significantly alter the aw values of the *mortadella* formulations, probably due to the high water content present in the product. However, a study conducted by Viuda-Martos et al. (2010) reduced the aw to 0.87 by adding 1% of orange dietary fibre, compared to the control sample, which had an aw of 0.89, attributed because the *mortadellas* in this study showed a lower moisture content (61.27–61.32%) [22].

##### Colour

Table 8 shows instrumental colour evaluation for *mortadellas* formulated with lemon dietary fibre. For lightness (L*) values, the treatment effects were statistically significant (*p* < 0.05), with higher L* values observed in samples containing higher concentrations of LDF. This indicates that the inclusion of LDF contributes to an increase in lightness, with the effect becoming more pronounced at higher concentrations. The redness (a*) significantly decreases with the addition of LDF (*p* < 0.05). The yellowness (b*) was affected by the addition of LDF at a concentration of 6%. The observed reduction in redness and the increase in yellowness can be attributed to the natural soft yellow hue of the LDF powder. Consequently, reformulated *mortadellas* appear paler than the control, as illustrated in Figure 2. This change reflects the influence of LDF on the visual properties of the final product, driven by its inherent colouration. The analysis of chroma (C*) and hue (h°) values in the *mortadella* samples suggests that incorporating LDF results in a product with a slightly yellowish colour and moderate chromatic intensity. These attributes indicate that the product appears visually appealing while maintaining a natural aspect, avoiding an artificial appearance.

Powell et al. (2019) [29], with the addition of 1% citrus fibre, reported colour coordinates similar to those observed in our study for alternatively cured all-pork bologna sausage under different lighting conditions, including retail display lights and darkness. This addition resulted in consistent L*, a*, and b* values across both dark and retail display lighting conditions. These findings suggest that the incorporation of citrus fibre contributes to stable colour attributes across various lighting environments [29]. Another study conducted by Gedikoğlu and Clarke (2019) aligns with our findings, reporting that the incorporation of citrus fibre in ground beef meatballs led to a decrease in L* and a* values, while the b* value increased with the addition of 10% lemon fibre [81].

#### 3.6.5. Textural Properties

The textural properties (TPA) of *mortadellas* are presented in Table 9. The incorporation of LDF significantly influenced the hardness of the samples (*p* < 0.05). However, no significant differences were observed between the 3% and 6% LDF samples. Hardness increased with higher concentrations of lemon dietary fibre. These results could considerably affect the sensory attributes of *mortadellas*, particularly for the LDF (6%), as greater hardness may make the product more challenging to consume and less appealing to consumers. Springiness was significantly affected only with the incorporation of 6% LDF in comparison to the control. In contrast, cohesiveness and chewiness were not significantly influenced by the addition of lemon dietary enriched fibre (*p* > 0.05). A recent study conducted by Sarıçoban and Unal (2022) examined the effect of orange albedo on the textural properties of fermented sausages [82]. The results of hardness align with this study, showing that higher concentrations of orange albedo (especially 2.5% and 5%) led to increased hardness. The control sample had the lowest hardness, while formulations with greater citrus-based powder incorporation exhibited a clear increasing trend in hardness.

#### 3.6.6. Residual Nitrite Level and Lipid Oxidation

**Figure 9:**
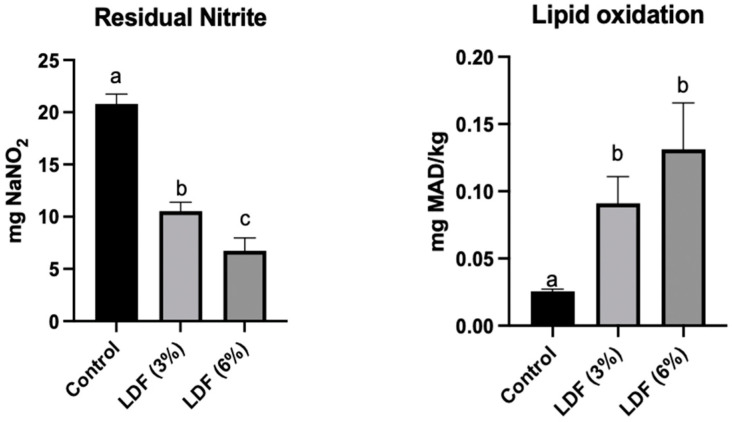
Residual nitrite (mg NaNO_2_/sample) and lipid oxidation (mg MDA/kg sample) of mortadellas formulated with LDF (3% and 6%). For each parameter, results followed by same case letter are not significantly different according to Tukey’s HSD post hoc test (*p* > 0.05). Data are presented as mean values of replications ± SD, where n = 3.

Figure 9 illustrates the interrelation between residual nitrite levels and lipid oxidation in reformulated *mortadellas*. Residual nitrite levels were significantly higher in the control sample, followed by LDF (3%) and LDF (6%) (*p* < 0.05). This research demonstrates that lemon dietary fibre effectively reduces residual nitrite levels, being a promising approach to lowering dietary nitrite intake. This reduction is particularly relevant in mitigating the potential formation of harmful n-nitroso compounds, known to carry carcinogenic, teratogenic, and mutagenic risks [83]. Conversely, lipid oxidation values revealed an inverse trend, with higher oxidation concentrations corresponding to lower residual nitrite levels. Lipid oxidation is a chemical process that deteriorates fats in meat, resulting in rancid flavours and unpleasant odours. This oxidation process is closely linked to the effects of nitrite, which is commonly used as a preservative in processed meats. Sodium nitrite plays a critical role in reducing TBARs (thiobarbituric acid reactive substances) values, which are lipid oxidation indicators, helping maintain meat quality over time. Lipid oxidation was minimal in the control, increasing in LDF (3%) and reaching the highest value in LDF (6%). The control sample differed significantly from the LDF (3%) and LDF (6%) samples (*p* < 0.05), but no significant difference was observed between the LDF (3%) and LDF (6%). Additionally, it is important to note that lipid oxidation levels in all reformulated *mortadellas* with lemon dietary fibre remained below the rancidity threshold (≥1.0). A study conducted by Karwowska et al. (2020) supports this observation, demonstrating that reducing the amount of sodium nitrite in the samples increased lipid oxidation [84]. Specifically, on day 0, the TBAR value was lower (0.43 mg/kg) in samples containing 150 mg/kg of nitrite compared to those without nitrite (0.88 mg/kg). Over the 15-day storage period, this trend was consistent, further emphasising the protective role of sodium nitrite against lipid oxidation in meat products. In addition, the biologically active compounds are not as effective as nitrites in decreasing lipid oxidation; however, the polyphenols found in fruit wines were responsible for the reduction in MDA levels [85].

### 3.7. Sensory Evaluation

**Figure 10:**
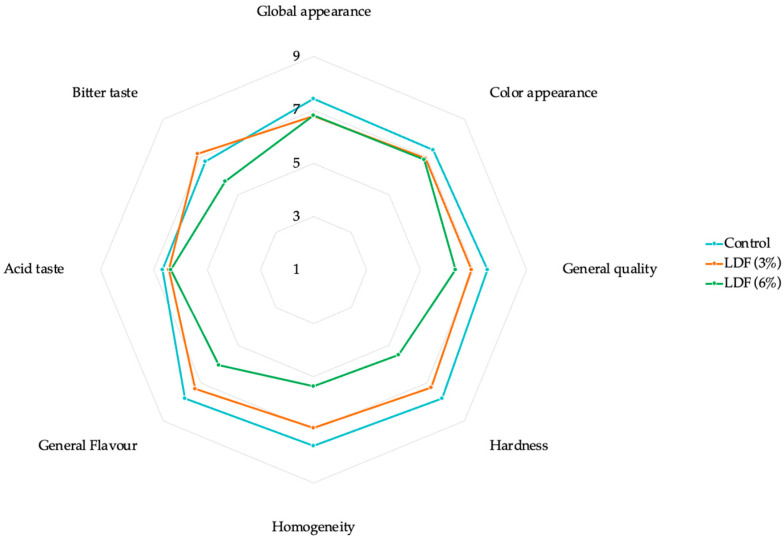
Sensory evaluation: A quantitative descriptive analysis was carried out on the different *mortadellas* reformulated with lemon dietary fibre (3% and 6%). Sensory score: 1—very unacceptable, 9—very acceptable.

Figure 10 presents the sensory evaluation of reformulated *mortadellas* containing 3% and 6% fibre. The parameters evaluated included global and colour appearance, general quality, hardness, homogeneity, general flavour, acid, and bitter taste. All parameters showed significant differences between conditions (*p* < 0.05). Notably, the most affected parameters were flavour, homogeneity, hardness, and bitter taste. *Mortadella* labelled as a “source of fibre” (LDF (3%)) was more acceptable to the panellists compared to those labelled as having a “high content of fibre” (LDF (6%)). Panellists detected the bitter taste, normally associated with citrus products, in *mortadellas* with LDF (6%). Additionally, the increased fibre content impacted hardness, with the control being rated the most favourable (7.82), followed by LDF (3%) (7.23) and LDF (6%) (5.52). Homogeneity was another parameter significantly (*p* < 0.05) influenced by LDF concentration. Panellists preferred the control (7.61), followed by LDF (3%) (6.93), with LDF 6% being the least favoured (5.35). These results suggest that future studies should focus on developing *mortadellas* with 3% fibre, as this formulation was well accepted and maintained better sensory qualities compared to higher fibre concentrations.

Other studies have evaluated the impact of adding lemon co-products on the sensory properties of food products. For instance, Fernández-Ginés et al. (2004) studied two types of albedo (raw and cooked) at five concentrations (0%, 2.5%, 5%, 7.5%, and 10%) in bologna sausages [32]. The formulations that achieved sensory properties closest to conventional sausages were those containing 2.5% and 5% raw albedo, as well as 2.5%, 5%, and 7.5% cooked albedo. Similarly, Fu et al. (2015) evaluated the incorporation of lemon fibre (LF) recovered from lemon pomace (0%, 3%, 6%, and 9% LF) in dough and Mantou (steamed bread) to assess its effects on colour, flavour, texture, and overall acceptability [54]. The most pronounced sensory impacts were observed in products formulated with 6% and 9% LF, indicating that higher concentrations of LF may decrease sensory acceptability.

## 4. Conclusions and Future Perspectives

Lemon co-products can be supplied as a potential functional and nutritional food ingredient containing abundant dietary fibre, as well as for their valuable functional properties, including technological and physicochemical benefits, along with preservation capabilities. The innovative LDF powder was obtained after the integrated extraction of (lemon hydrolat, essential oil, phenolic compounds extract, and pectin), which contains notable phenolic (free and bound) compounds that provide antioxidant properties and help reduce bacterial growth. These combined attributes make such powders highly suitable for selection as ingredients in the development of new food products. The incorporation of lemon dietary fibre (LDF) in *mortadellas* significantly influenced the colour, texture, and mineral composition of the products. Higher levels of LDF result in a paler colour and increased hardness in *mortadellas.* Additionally, LDF contributes to a reduction in sodium levels, which is highly beneficial for global population health. Furthermore, LDF reduces residual nitrite levels, though a slight increase in lipid oxidation accompanies this reduction; however, it remained below the rancidity threshold (≥1.0) in all products, ensuring acceptable product quality. Sensory panellists evaluated the lemon dietary fibre-enriched *mortadellas* positively, demonstrating a distinct preference for the LDF (3%) formulation. In conclusion, this study highlights the potential of repurposing lemon co-products to develop value-added processed meat products with 3% and 6% LDF, which can be classified as “source of fibre” and “high in fibre”, respectively. Additionally, further studies are recommended to assess the shelf-life of these products during storage, focusing on microbiological stability, texture, colour, lipid oxidation, and residual nitrite levels to ensure long-term quality and safety.

## Figures and Tables

**Table 1:** Proximate composition (g/100 g sample) of lemon dietary fibre-based powder.

	LDF
Moisture (%)	3.8 ± 0.0
Protein (%)	4.6 ± 0.0
Lipids (%)	0.0 ± 0.0
Ash (%)	1.2 ± 0.0
Carbohydrates (%)	90.5 ± 0.0
Total Dietary Fibre (TDF) (%)	85.8 ± 1.1
Insoluble Dietary Fibre (IDF) (%)	52.6 ± 0.7
Soluble Dietary Fibre (SDF) (%)	33.2 ± 0.8
Cellulose (as glucose)	9.6 ± 0.9
Hemicellulose	6.8 ± 0.4
Soluble Lignin	9.9 ± 0.4
Insoluble Lignin	5.6 ± 0.4

The values are expressed as g/100 g DW (dry weight), where n = 3.

**Table 2:** Minerals composition (mg/100 g; n = 3) of lemon dietary fibre-based powder.

	LDF (mg/100 g)
Calcium	425.96 ± 4.54
Phosphorus	184.60 ± 7.27
Potassium	77.92 ± 6.53
Magnesium	15.31 ± 1.60
Sodium	9.92 ± 1.33
Iron	1.28 ± 0.46
Copper	0.62 ± 0.01
Zinc	0.58 ± 0.27
Manganese	0.17 ± 0.00

**Table 3:** Physicochemical [pH, water activity (a_w_) (n = 3), and colour coordinates (n = 6) (L*, lightness; a*, red/green coordinate; b*, yellow/blue coordinate; C*, chrome; h°, hue)] of lemon dietary fibre-based powder.

	LDF
pH	3.27 ± 0.01
a_w_	0.424 ± 0.02
L*	77.91 ± 1.35
a*	0.69 ± 0.08
b*	13.59 ± 0.38
C*	13.74 ± 0.37
*h°*	86.89 ± 0.43

**Table 4:** Phenolic compound quantification by HPLC-DAD of LDF in free phenolic extracts (FP), bound phenolic extracts obtained by alkaline hydrolysis (BP-ALK), and by acid hydrolysis (BP-ACD).

Phenolic Compound	RT (min)	Free Phenolics (FP)		Bound Phenolics (BP-ALK)		Bound Phenolics (BP-ACD)		
		MeOH	H_2_O	MeOH	H_2_O	MeOH	H_2_O	
		(mg/100 g DW)						
Vanillin	22.02			9.16 ± 2.14 ^a^	5.90 ± 1.80 ^b^			
Eriocitrin	23.42	146.35 ± 0.81 ^a^	68.75 ± 4.23 ^b^					
p-coumaric acid	23.99			7.06 ± 0.33 ^a^	5.47 ± 1.70 ^b^			
Ferulic acid	25.80			5.43 ± 0.55 ^a^	3.93 ± 0.19 ^b^			
Apigenin-7-O-Glucoside	29.02			8.82 ± 1.84				
Hesperidin	29.24	894.44 ± 4.63 ^a^	89.97 ± 1.39 ^b^					
Rutin	29.55			3.41 ± 0.22				
Luteolin-7-O-Glucoside	30.27			7.43 ± 1.93				
Hesperetin	48.11					5.30 ± 0.21 ^a^		6.00 ± 0.19 ^a^

Different lowercase letters in same row indicate significant differences (*p* < 0.05), as determined by Tukey’s Multiple Range Test. Data are presented as mean ± SD, where n = 3.

**Table 5:** Antibacterial activity of lemon dietary fibre-based powder.

		Log_10_ (CFU/mL)	Log Reduction
*Gram-positive*	*S. aureus* Control	9.5 ± 0.0 ^a^	-
	*S. aureus* LDF (3%)	9.2 ± 0.0 ^b^	0.3 ± 0.1
	*B. cereus* Control	9.8 ± 0.0 ^a^	-
	*B. cereus* LDF (3%)	Total inhibition ^b^	
*Gram-negative*	*E. coli* Control	9.6 ± 0.0 ^a^	-
	*E. coli* LDF (3%)	9.5 ± 0.1 ^b^	0.1 ± 0.0
	*S. enterica* Control	9.9 ± 0.0 ^a^	-
	*S. enterica* LDF (3%)	9.9 ± 0.0 ^a^	0.0 ± 0.0

Values followed by the same letter within the same column are not significantly different (*p* > 0.05), according to the *t*-test (non-parametric test) (*p* > 0.05). Data are presented as the mean values of replications ± SD, where n = 3.

**Table 6:** Chemical composition of *mortadellas* formulated with lemon dietary fibre (3% and 6%).

Formulation	Moisture (%)	Fat (%)	Protein (%)	Ash (%)
Control	66.48 ± 0.00 ^a^	11.94 ± 0.05 ^a^	14.75 ± 0.44 ^a^	3.78 ± 0.00 ^a^
LDF (3%)	63.65 ± 0.02 ^ab^	11.75 ± 0.57 ^a^	14.88 ± 0.53 ^a^	3.46 ± 0.00 ^a^
LDF (6%)	62.15 ± 0.00 ^b^	11.32 ± 0.26 ^a^	15.15 ± 1.26 ^a^	3.53 ± 0.00 ^a^

Control: *mortadella* without lemon by-products; LDF 3%: *mortadella* formulated with LDF at 3%; LDF 6%: *mortadella* formulated with LDF at 6%. Values followed by same letter within same column are not significantly different (*p* > 0.05), according to Tukey’s HSD post hoc test (*p* > 0.05). Data are presented as mean values of replications ± SD, where n = 3.

**Table 7:** pH and aw values of *mortadellas* formulated with lemon dietary fibre (3% and 6%).

Formulation	pH	a_w_
Control	6.02 ± 0.02 ª	0.965 ± 0.00 ^a^
LDF (3%)	5.66 ± 0.03 ^b^	0.965 ± 0.00 ^a^
LDF (6%)	5.36 ± 0.03 ^c^	0.963 ± 0.00 ^a^

Control: *mortadella* without lemon by-products; LDF 3%: *mortadella* formulated with LDF at 3%; LDF 6%: *mortadella* formulated with LDF at 6%. Values followed by same letter within same column are not significantly different (*p* > 0.05), according to Tukey’s HSD post hoc test (*p* > 0.05). Data are presented as mean values of replications ± SD, where n = 3.

**Table 8:** Colour coordinates of *mortadellas* formulated with lemon dietary fibre (3% and 6%).

Formulation	L*	a*	b*	C*	*h°*
Control	70.93 ± 0.79 ^a^	6.96 ± 0.11 ^a^	10.79 ± 0.20 ^a^	12.84 ± 0.16 ^a^	57.17 ± 0.73 ^a^
LDF (3%)	73.41 ± 0.93 ^b^	5.79 ± 0.21 ^b^	12.45 ± 1.23 ^a^	13.59 ± 1.08 ^a^	66.29 ± 2.54 ^b^
LDF (6%)	73.96 ± 0.93 ^b^	5.43 ± 0.24 ^b^	14.33 ± 0.57 ^b^	15.46 ± 0.56 ^b^	68.00 ± 0.97 ^b^

Control: *mortadella* without lemon by-products; LDF 3%: *mortadella* formulated with LDF at 3%; LDF 6%: *mortadella* formulated with LDF at 6%. Values followed by same letter within same column are not significantly different (*p* > 0.05), according to Tukey’s HSD post hoc test (*p* > 0.05). Data are presented as mean values of replications ± SD, where n = 9.

**Table 9:** Textural properties (TPA) of *mortadellas* formulated with lemon dietary fibre (3% and 6%).

Formulation	Hardness (N)	Cohesiveness	Springiness (mm)	Chewiness (N mm)
Control	33.79 ± 0.85 ^a^	0.27 ± 0.03 ^a^	0.39 ± 0.04 ^a^	3.48 ± 0.60 ^a^
LDF (3%)	41.40 ± 2.57 ^b^	0.23 ± 0.03 ^a^	0.33 ± 0.03 ^ab^	3.57 ± 0.21 ^a^
LDF (6%)	45.10 ± 2.65 ^b^	0.24 ± 0.02 ^a^	0.30 ± 0.01 ^b^	2.45 ± 0.34 ^a^

Control: *mortadella* without lemon by-products; LDF 3%: *mortadella* formulated with LDF at 3%; LDF 6%: *mortadella* formulated with LDF at 6%. Values followed by same letter within same column are not significantly different (*p* > 0.05), according to Tukey’s HSD post hoc test (*p* > 0.05). Data are presented as mean values of replications ± SD, where n = 6.

## Data Availability

The data presented in this study are available on request from the corresponding author due to privacy.
